# Intratubular penetration capacity of HiFlow bioceramic sealer used with warm obturation techniques and single cone: A confocal laser scanning microscopic study

**DOI:** 10.1016/j.heliyon.2022.e10388

**Published:** 2022-08-27

**Authors:** Alberto Casino Alegre, Susana Aranda Verdú, José Ignacio Zarzosa López, Eliseo Plasencia Alcina, Jorge Rubio Climent, Antonio Pallarés Sabater

**Affiliations:** a Department of Endodontics and Restorative Dentistry, School of Medicine and Dentistry, Catholic University of Valencia, Quevedo 2, 46001, Valencia, Spain; b Doctoral School, Catholic University of Valencia, 46001, Valencia, Spain

**Keywords:** Bioceramic sealer, Confocal laser scanning microscopy, Tubule penetration, Warm gutta-percha techniques, Single cone

## Abstract

**Objective:**

The aim of this paper was to evaluate the intratubular penetration area of a bioceramic sealer, using continuous wave (CW), vertical condensation (VC) with two different types of gutta-percha (conventional (NG) and bioceramic-coated (BG)) and single cone (SC) technique with BG gutta-percha, in different root thirds.

**Methods:**

A total of 150 mature single-root human teeth (including incisors, canines and premolars) were prepared and randomly divided into five groups (n = 30). Teeth were filled using a bioceramic sealer (TotalFill BC Sealer HiFlow ®) and two different types of gutta-percha, with CW and VC techniques, the teeth in the control group were filled with SC technique and BG gutta-percha. The teeth were sectioned and evaluated as one-third portions in each case under a confocal laser microscope. The penetration area measurements were carried out with the Autocad ® programme. Data was analyzed using the one-factor ANOVA test (p < 0.05) and Post Hoc Test (p < 0.05).

**Results:**

The ANOVA Test showed significant differences in the penetration areas of the five obturation techniques (P < 0.05). The Post Hoc Test exhibited significant differences in multiple comparisons (P < 0.05). There was more dentinal tubule penetration in the coronal third than in the apical third in all techniques.

**Conclusions:**

The intratubular penetration of the bioceramic sealer was influenced by the obturation techniques tested, but not by the different gutta-percha tested. There was more penetration of sealer in the warm obturation techniques than the SC, regardless of the type of gutta-percha used.

## 1 Introduction

After chemomechanical preparation of the root canal in an endodontic procedure, the presence of microorganisms was detected [1, 2]. It is essential to prevent reinfection by creating an airtight seal of the canal system using obturation materials [3]. The use of a sealer is essential because it establishes a bond between the gutta-percha and the root dentine [4].

Bioceramic sealer penetration into dentinal tubules is essential in order to create a mechanical anchorage between the sealer and the dentinal tubules [5] and chemical hydroxyapatite formation [6]. In addition, it is used to eliminate biofilms and residual microorganisms either by contact action or by burial in the dentinal tubules [7, 8].

Previously, the usual root canal filling practice was to apply a considerable amount of gutta-percha and a small proportion of sealer [9]. Kim et al. [10] showed that the filling with bioceramic sealers used in warm obturation techniques required higher volumes of gutta-percha than single cone (SC) techniques. However, bioceramic sealers were manufactured for cold obturation techniques, in particular for the use in a SC technique [11]. A relatively high sealer proportion was no longer thought a disadvantage, as the biological and antibacterial properties of bioceramic sealers are believed to improve the success of the endodontic treatment. Due to the low condensation pressure of the gutta-percha and calcium silicate-based sealer during SC obturation, this technique is considered incapable of adequately filling any complicated root canal anatomy [12]. Warm obturation techniques were consequently developed to allow a better three-dimensional obturation of root canal anatomy [12, 13], by heating and condensing the gutta-percha we achieve a better adaptation to the walls of the canal [9]. This results in a lower amount of sealer in the canal obturation [9]. However, the sealer is of paramount importance in sealing the dentinal tubules [14].

EndoSequence BC Sealer HiFlow® (HiFlow) (Brasseler USA®, Savannah, GA, USA) is a bioceramic sealer developed to be heat-resistant [15]; it is similar to the EndoSequence BC Sealer® (BC Sealer) but with some modifications in its composition that make it more suitable for warm obturation techniques. HiFlow® has a lower viscosity than the original BC Sealer formulation. For an adequate setting of the sealer, the right level of humidity of the dentinal tubules is essential since the setting reaction of bioceramic materials is a process requiring several weeks [16]. The use of warm obturation technique maybe affect the chemophysical properties of the sealer and disturb the setting reaction. For this reason, it is essential to have a good grounding in the use of root obturation techniques with these bioceramic sealers. So far, no data is available addressing the long-term effects of heat treatment on bioceramic sealers [9].

EndoSequence BC Sealer® and EndoSequence BC Sealer HiFlow® have the same composition as TotalFill BC Sealer® (BC Sealer) (FKG Dentaire SA, La-Chaux-de-fonds, Switzerland) and TotalFill BC Sealer HiFlow® (HiFlow) [17]. The composition of the HiFlow premixed calcium silicate–based sealers are made up of zirconium oxide, tricalcium silicate, dicalcium silicate, calcium hydroxide and fillers [18].

Recently, the behaviour of other bioceramic sealers, such as BC Sealer or BioRoot RCS® (BR; Septodont, St. Maur-des-Fossés, France) has been investigated after exposure to heat. The chemophysical properties were investigated during or shortly after heat exposure [19, 20, 21, 22, 23]. While the physical properties of HiFlow were not adversely affected by heat, an increase in viscosity of the BioRoot RCS® and BC Sealer was found [22]. iRoot SP® (Innovative BioCeramix Inc., Vancouver, Canada) resulted in a reduced flow [24].

In the early 2000s, specialized manufacturers introduced root obturation techniques with the “monoblock” concept in which the gutta-percha, the sealer and the dentin generated a single unit [25]. Currently, there are few articles evaluating the penetration capacity of bioceramic sealer with warm obturation techniques using different types of gutta-percha. It seems that the design and development of bioceramic gutta-percha is aimed at the SC technique forming a “monoblock” obturation system in that it has a very similar composition to the gutta-percha and the bioceramic sealer. Most of the studies evaluated the likelihood of failure of these two materials, i.e. whether they separate when a certain force is applied, categorizing the failures as different types, namely: adhesive, cohesive and mixed types [17, 26].

The TotalFill BC filling system consists of TotalFill BC Points® bioceramic-coated (BG) gutta-percha and BC Sealer; the obturation system makes use of the moisture naturally present in the canal to start the setting reaction [27]. BC Sealer is biocompatible, osteogenic and offers zero shrinkage [28] and has even been observed to expand [6]. According to the manufacturer, the objective when the obturation system (TotalFill BC Points® and BC Sealer) is used with the SC, a fissure-free seal is created.

There is a general lack of information available concerning the capacity of tubule penetration of BC Sealer when used together with gutta-percha tips with different compositions (bioceramic or conventional) and different tapers (0.2–0.4-0.6–0.8).

The aim of this paper was to evaluate the intratubular penetration area of a calcium silicate-based sealer (HiFlow), using two warm obturation techniques, continuous wave (CW) and vertical condensation (VC) with two different types of gutta-percha (conventional (NG) and BG) and also SC technique with BG in different root thirds. The null hypothesis asserts there are no differences between the penetration areas obtained for each of the obturation techniques.

## 2 Materials and methods

This piece of research was approved by the Research Ethics Committee of UCV, (Registration number: UCV/2019-2020/001.).

### 2.1 Selection of samples

To carry out the study, 150 human teeth with a single root were selected (including incisors, canines and premolars). The teeth were extracted for periodontal reasons. Roots with acute curvatures, immature apex, resorption, previous endodontic treatment, calcification, fractures or initial apical sizes larger than 15 were rejected. After extraction, the teeth were immersed for one hour in a 5.25% sodium hypochlorite solution (NaOCl) after which the root surfaces were cleaned with a Gracey® 1-2 curette (Hu-Friedy, USA) and then stored in a saline solution.

### 2.2 Root canal preparation

Two preoperative X-rays were taken in two views to check the presence of a single canal. Buccolingual and mesiodistal parallel radiographs were obtained for each tooth. After opening the root canal system with a tapered cone burr (Komet, Lemgo, Germany) and constant irrigation, the canal was located with a DG16® endodontic probe (Hu-Friedy, USA). The root of the clinical crown was separated at the amelocemental junction with a handpiece diamond disc and water cooling; a size 10 or 15 K file was then introduced into the canal space, the working length (WL) was established 0.5 mm from the apical foramen by visual observation.

All canals were prepared with Protaper Gold® (Dentsply Maillefer, Ballaigues, Switzerland) according to the producer’s instructions. The shaping files S1 (250 rpm and 3 Ncm) and S2 (250 rpm and 1 Ncm) were used with circumferential movements and brushing at the working length, while the finishing files F1 (250 rpm and 1.5 Ncm) and F2 (250 rpm and 2.5 Ncm), were used with a pecking motion with the Gold Reciproc™ motor (VDW, Munich, Germany). After each file was used, the canal was flushed out with 5.25% NaOCl solution. The permeability of the canals was checked by inserting a size 10 file through the apical foramen after instrumentation was completed.

As the final irrigation protocol, canals were irrigated for 1 min with 5 ml of 5.25% NaOCl, 1 min with 5 ml of 17% EDTA, and 30 s with 5 ml of chitosan-hydroxyapatite precursor, 10 ml of saline solution was used for a final flush out and also used in the established order of different irrigants [29, 30]. The irrigants were activated using the EDDY® sonic tip system (VDW, München, Germany) with Air Scaler. The canals were dried with F2 paper tips. This chemomechanical sample preparation procedure was the common denominator, regardless of the obturation technique used.

### 2.3 Obturation of the root canals

0.1% of Rhodamine B™ (Sigma-Aldrich Corp., USA) was added to the bioceramic sealer in relation to the weight for its subsequent observation through the confocal laser microscope, thanks to the fluorescent property of the dye.

The samples were then randomly divided into 5 experimental groups (n = 30). The samples were sealed with the different obturation techniques set forth as follows (Table 1).

**Table 1:** Groups and Description of the Study Design.

Number	Technique	Temperature	Sealer	Gutta-percha	Unit Obturation	Explanation
1.	Continuous wave technique	Hot plugger: 220 °C Warm gutta-percha injection unit: 200 °C	HiFlow	Protaper F2® gutta-percha, conventional gutta-percha pellets	E&Q Master® (Meta Biomed, Chalfont, PA, USA).	The teeth were filled using the technique designed by Buchanan. The plugger was checked with the rubber stopper positioned at less than 4 mm from the working length.
2.	Continuous wave technique	Hot plugger: 220 °C Warm gutta-percha injection unit: 200 °C	HiFlow	TotalFill BC Points® bioceramic-coated gutta-percha 25 (0.6), bioceramic gutta-percha pellets	E&Q Master® (Meta Biomed, Chalfont, PA, USA).	The teeth were filled using the technique designed by Buchanan. The plugger was checked with the rubber stopper positioned at less than 4 mm from the working length.
3.	Vertical condensation technique	Hot plugger: 100 °C Warm gutta-percha injection unit: 200 °C	HiFlow	Protaper F2® gutta-percha, conventional gutta-percha pellets	The System-B® obturation unit (Sybron Dental, Orange, CA, USA)	The teeth were filled using the technique designed by Schilder. The hot plugger was used to remove 2–3mm portions of gutta-percha and condensing it until reaching 4mm of the working length.
4.	Vertical condensation technique	Hot plugger: 100 °C Warm gutta-percha injection unit: 200 °C	HiFlow	TotalFill BC Points® bioceramic-coated gutta-percha 25 (0.6), bioceramic gutta-percha pellets	The System-B® obturation unit (Sybron Dental, Orange, CA, USA)	The teeth were filled using the technique designed by Schilder. The hot plugger was used to remove 2–3mm portions of gutta-percha and condensing it until reaching 4mm of the working length.
5.	Single cone	Hot plugger: 230 °C	HiFlow	TotalFill BC Points® bioceramic-coated gutta-percha 25 (0.6)	E&Q Master® (Meta Biomed, Chalfont, PA, USA).	The gutta-percha cone was inserted in the canal at working length with the sealer. It was then seared off with the hot plugger in the coronal third and compacted.

### 2.4 Specimen preparation

**Figure 1:**
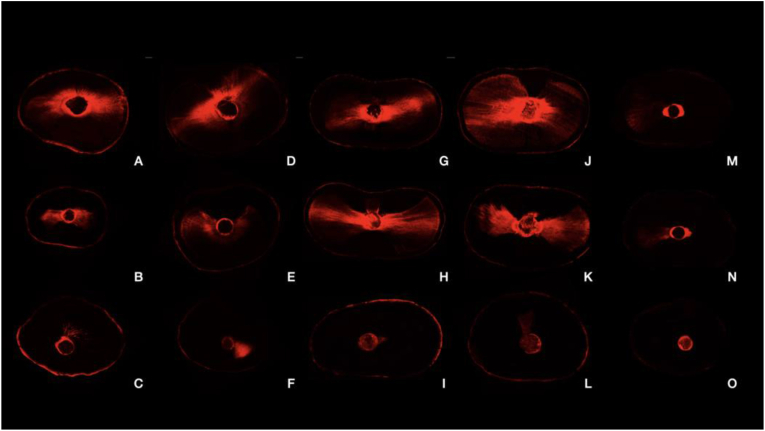
Continuous wave technique normal gutta-percha: A (coronal), B (middle), C (apical). Continuous wave technique bioceramic gutta-percha: D (coronal), E (middle), F (apical). Vertical condensation technique normal gutta-percha: G (coronal), H (middle), I (apical). Vertical condensation technique bioceramic gutta-percha: J (coronal), K (middle), L (apical). Single Cone: M (coronal), N (middle), O (apical).

Once all the samples were sealed, they were stored at 37 °C and 100% humidity in a laboratory incubator for 14 days to allow complete sealer setting. The root was divided into three parts at the dental laboratory of Catholic University of Valencia, taking a sample from each third: the coronal, middle and apical third (the apical third was taken by subtracting a length of two millimetres from the root apex). Horizontal cuts were made using a 0.3 mm diamond disc handpiece with water cooling [31], 1 mm thick slices were then obtained; the slices were polished with Soft Lex discs (3M (™) ESPE (™) St. Paul, MN, USA). After observation with the confocal laser microscope (Leica TCS SP8 Confocal Microscope) at the University of Valencia (unit of Central Service for Experimental Research (SCSIE)) and the 5x object lens, photographs of each of the samples were taken for analysis and studied (Figure 1).

**Figure 2:**
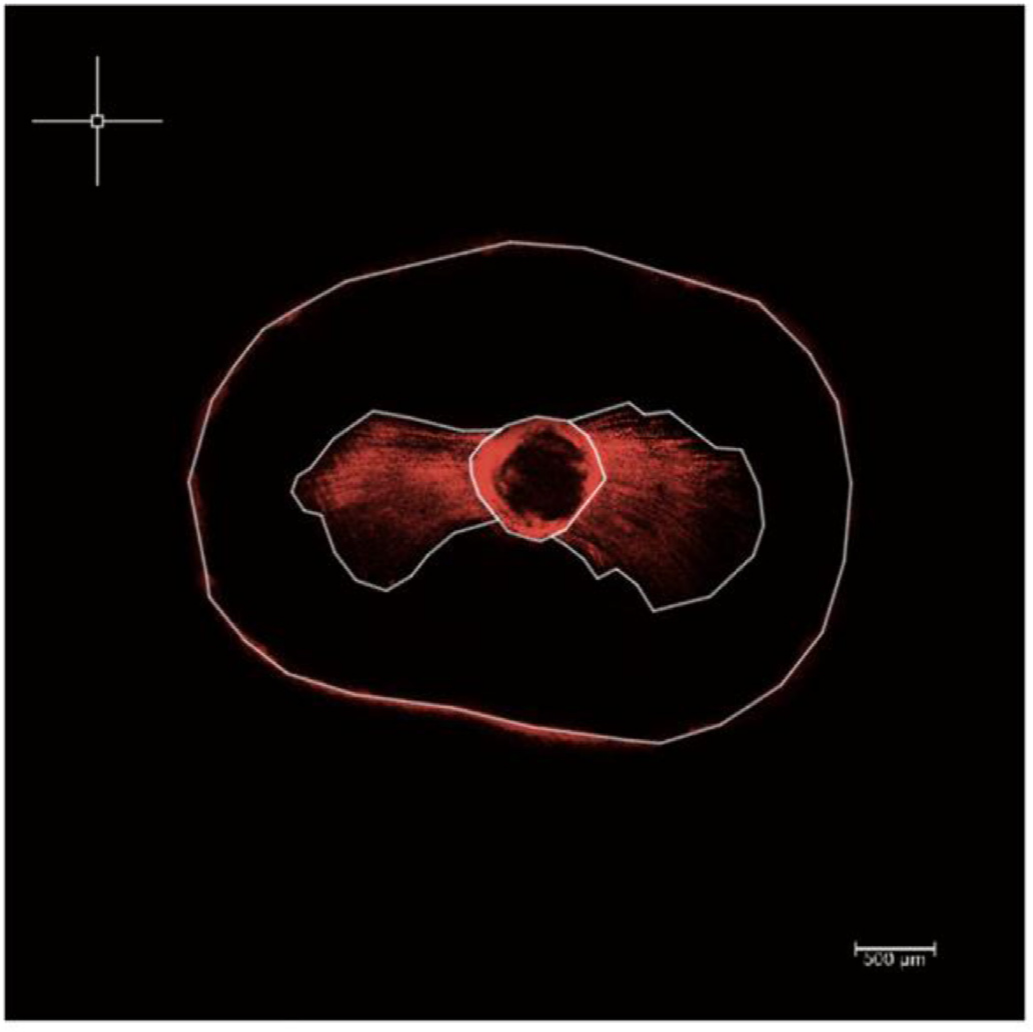
The white lines outline the tooth, the root canal, and penetration within the root canal as detected by the AutoCad Software.

The measurements of the penetration areas of the sealer were carried out with AutoCad® Software from the images obtained and collected in a data sheet. Fist, each image was scaled to 500 μm in order to obtain a correct measurement of all its elements. The appropriate AutoCad tool function was applied to the area of tubular sealer penetration and the canal area, to obtain the penetration area (Figure 2). The penetration area was calculated by adding the sealer penetration area in the tubules plus the canal area (mm^2^).

All measurements were recorded by one of the authors. In case of doubt on first viewing, the sample was polished, and a new image was then obtained for analysis. All data was recorded, and then analysed.

### 2.5 Statistical analysis

**Figure 3:**
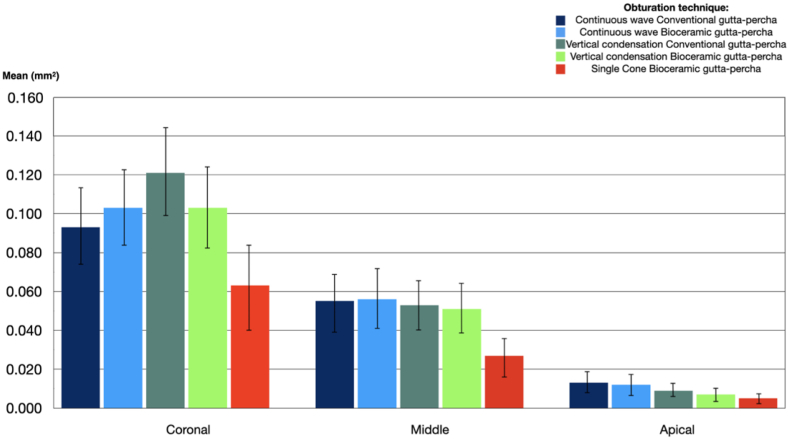
Mean area penetration (mm^2^), third portion assessed and obturation technique.

The statistical analysis of the data collected for the present study was carried out using SPSS 23 software using a confidence level of 95% and considering them statistically significant (p < 0.05). As the sample size is sufficiently large, (n = 30), we used parametric methods of comparison. The one-way ANOVA test was used to compare means and to determine the statistical and significant effect of the study variables (obturation techniques, heat and penetration of dentinal tubules) (Table 2 and Figure 3). The Post Hoc test revealed differences between groups (Table 3).

**Table 2:** Penetration area of the sealer, showing means and *p* values (ANOVA Test).

Third	Obturation method	Mean (mm^2^)	P value (p < 0.05)
Coronal	CW-BG	0.103 ± 0.019	0.002
	CW- NG	0.093 ± 0.019	
	VC-BG	0.103 ± 0.021	
	VC-NG	0.121 ± 0.022	
	SC-BG	0.062 ± 0.022	
Middle	CW-BG	0.056 ± 0.015	0.005
	CW- NG	0.055 ± 0.014	
	VC-BG	0.051 ± 0.013	
	VC-NG	0.053 ± 0.013	
	SC-BG	0.027 ± 0.009	
Apical	CW-BG	0.012 ± 0.005	0.005
	CW- NG	0.013 ± 0.005	
	VC-BG	0.007 ± 0.003	
	VC-NG	0.009 ± 0.003	
	SC-BG	0.005 ± 0.002

**Table 3:** Post Hoc Test *p* values.

Third	Obturation method	Obturation method	P value (p < 0.05)
Coronal	SC-BG	CW-BG	0.058
		CW-NG	0.393
		VC-BG	0.051
		**VC-NG**	**0.001**
Middle	SC-BG	**CW-BG**	**0.010**
		**CW-NG**	**0.017**
		VC-BG	0.078
		**VC-NG**	**0.035**
Apical	SC-BG	**CW-BG**	**0.045**
		**CW-NG**	**0.031**
		VC-BG	0.816
		VC-NG	0.174

## 3 Results

In all thirds, the ANOVA test showed significant differences between at least two of the obturation techniques (p < 0.05) (Table 2 and Figure 3).

In the coronal third, the Post Hoc test showed there was a statistical difference between the SC and VC with NG (p = 0.001), the penetration average was lower using the SC technique. There was no statistical difference observed compared to the other techniques (Table 3).

In the middle third, the Post Hoc test revealed there was a statistical difference between the SC and CW with NG (p = 0.017), CW with BG (p = 0.035) and VC with NG (p = 0.001). In this third, the penetration average was lower in the SC. There was no statistical difference compared to the other techniques (Table 3).

In the apical third, the Post Hoc test exhibited there was a statistical difference between the SC and CW with NG (p = 0.031), CW with BG (p = 0.045), the penetration average was lower in the SC. There was no statistical difference compared to the other techniques (Table 3).

## 4 Discussion

During the study, we used the confocal laser microscope, since the conditioning of the specimens for the scanning electron microscope needs to be dried [32]. The samples had to be subjected to gold-palladium sputtering and manipulated under vacuum [33]. This whole series of procedures can lead to loss of sealer quantity, deformation of the sample and of the materials to be studied [34], which can be responsible for the production of artefacts, compromising an adequate evaluation compared to confocal laser microscopy analysis [35, 36].

Rhodamine B could be suitable with the bioceramic sealers [8], because the small amount (0.1%) used did not modify the sealer’s qualities [37]. For this reason, it was the dye of choice in our research. The sample cuts were performed in the horizontal plane, as the dentine of the root canal cannot be completely observed in the longitudinal plane [38].

The correct evaluation of the samples in the microscope was difficult because the precision cuts and an adequate level of polishing were essential. The principal problem was one of accuracy: the root cuts had to be 1 mm deep, thin and completely straight. The samples of the apical third were particularly difficult to manipulate because of their size.

The penetration of a sealer is vitally important and it may be influenced by several factors such as the chemophysical properties of the sealer, the obturation technique and the anatomy of the root canal system [34]. Additionally, the variations in instrumentation, irrigation techniques and in the irrigants themselves play an essential role in the penetration of the sealer in the dentin tubules. These factors are influential in the removal of residual smear layer, tissue or debris. In our study we use a protocol with 5.25% NaOCl, 17% EDTA, and chitosan-hydroxyapatite precursor, with sonic activation to prepare the dentine surface for greater dentinal penetration of bioceramic sealer. Hashmi et al. [39] observed that the chitosan-hydroxyapatite precursor, enhances dentin surface wettability to facilitate greater bioceramic sealer penetration in the dentin. The irrigant used in this study to effectively remove the smear layer was characterized by the use of 17% EDTA and sonic activation, in accordance with the study of Virdee et al. [40].

The viscosity and flow of endodontic sealers are important in determining the effectiveness that they penetrate in the dentinal tubules [15]. The influential factors for the viscosity of the sealer include particle size, temperature and setting time [41, 42, 43]. In a study by Zhou et al. [44], BC sealer exhibited a higher flow than the epoxy resin-based sealers at room temperature. Chen et al. showed that HiFlow had a lower viscosity than the BC sealer at different temperatures [20]. The fine particle size (<1 μm) is one of main reasons why the deep diffusion is more likely to occur in the bioceramic sealers. In addition to their increase in volume during the setting and their basic pH, there was a resulting denaturing of the collagen fibres, resulting in tubular penetration [6, 7, 45].

The warm obturation techniques with bioceramic sealers have become a controversial theme. There were significant changes in the properties of the bioceramic sealers after heating during the thermoplasticized obturation techniques [18]. Apatite-forming capacity is another desirable property in calcium silicate sealers, but the temperature increase may affect the biomineralization process [46]. Therefore, we believe it is essential to know the chemomechanical properties of bioceramic sealers and how they may be affected by the application of heat. Additionally, we must also be aware of the instructions given by the manufacturers regarding the bioceramic sealers and the recommended obturation technique.

Chen et al. compared [20] two bioceramic sealers (BC Sealer and HiFlow) and how heat action may influence different properties. The heat application in HiFlow did not considerably modify the setting time, micro hardness, solubility, chemical composition and cytotoxicity. They concluded HiFlow gave a better performance on flow/viscosity and film thickness than BC sealer, especially under high temperatures. When the temperature is increased, the sealer HiFlow has a shorter setting time [18, 20]. These specific properties of HiFlow may be one of the reasons why in our study we observed greater tubular penetration when applying heat-based rather than cold techniques. Therefore, HiFlow is an adequate sealer in order to use with the warm obturation techniques and according to the manufacturer it was designed for use with high temperatures up to 220 °C.

It is commonly believed that increasing the pressure in the warm obturation techniques produces a significant level of sealer penetration in the dentin tubules, however the literature demonstrates contradictory results [15]. For these inconsistent outcomes five different obturation techniques were studied, four warm obturation techniques and one cold technique.

We used one group of SC with BG points as the manufacturers recommended this type of BG points and bioceramic sealer with the SC technique [6, 47]. They suggest that the sealing ability should be improved by joining the sealer and gutta-percha with the same bioceramic particles [48]. This chemo-mechanical union creates a junction that may function like a “tertiary monoblock”. The obturation materials should have an elastic modulus close to dentin in order to reinforce the root [25]. Although Osiri et al. [26] showed that with a much lower elastic modulus than of dentin, the bioceramic sealer with BG could enhance the fracture resistance of the prepared roots. In addition, the adhesion to root dentin plays a major role in reinforcing the prepared roots rather than the elastic modulus. These findings were not consistent with the monoblock concept that Tay et al. [25] proposed, in that the elastic modulus of material is an important factor for increased fracture resistance of the roots. The elastic modulus of BG was 0.20 ± 0.03 GPa, BC sealer 2.54 ± 0.13 GPa; dentine 8.60 ± 0.86 GPa respectively [26]. In general the gutta-percha (NG or BG) innately lacks adhesion to dentin *per se* [25]. Al-Hiyasat et al. [17] used the bioceramic sealer with BG with the SC technique and they obtained a sealer junction and BG that is better than the resin sealer and NG with the SC technique. Due to these arguments, we used only one group of SC with BG.

Our results showed a greater degree of penetration in the warm obturation techniques versus SC in all thirds, the area of penetration for each technique was greater in the coronal third compared to the apical portion. These results are in agreement with the those of previous studies [8, 15, 26]. Furthermore, we observed a lower degree of tubule penetration area in the apical third as regarding the middle and coronal third. Different studies have revealed that the depth of sealer penetration varies in each one thirds of the root canal [7, 49, 50, 51]. This may be due to the size and density of the tubule, as the size of the tubule decreases towards the apex [52] and the exchange of irrigants, as it becomes increasingly difficult as we move towards the apex [15]. The differing pressures in the different obturation techniques, the heat of the pluggers applied and the chemophysical properties of the sealer, all have a decisive influence on sealer penetration. On average, HiFlow has a particle size of 0.2 μm. This feature might improve its penetration into dentin tubules [26], notably in the cramped tubules in the apical third. In our opinion, these reasons may explain the much lower degree of sealer penetration in the apical third rather than the coronal third.

In the study by Eymirli et al. [38], the researchers evaluated the penetration ability of the BC Sealer in three outcomes groups: obturation with sealer only, sealer plus bioceramic gutta-percha .02 and sealer plus bioceramic gutta-percha .04 with the SC technique. Significantly greater sealer penetration area was achieved when the sealer was used with a BG .04, whereas there was no difference between the sealer and BG .02 groups. The use of a gutta-percha point with an adequate taper that fits snugly to the prepared canal shape generates some pressure that would enhance dentinal tubule penetration of the evaluated bioceramic sealer. With a fitting master cone to the master file, it is possible to minimize the amount of sealer, which decreases the for gaps. This pressure level in the SC technique is lower than in the warm obturation techniques, and this factor may be one of the differences in the dentinal tubule penetration sealer as we observed in our study. This disparity could improve the dentinal tubules penetration of the sealer during the obturation in the warm obturation techniques in all thirds [8]. Due to the compression in the warm obturation techniques, the sealer is pressed at the periphery of the canal, producing a thin layer of sealer on the dentinal wall [53].

Osiri et al. [26] studied the penetration capacity of bioceramic sealer with SC and BC Sealer with BG and AH Plus® with NG, obtaining greater penetration of the bioceramic group; furthermore, greater penetration was observed from coronal to apical as in our study. Turkel et al. [54], compared AH Plus and BC Sealer with NG and the SC technique, and they showed that the calcium silicates-based sealer had greater tubule penetration than the resin sealer. These results are in accordance with the aforementioned studies [7, 55]. This differences in the results may be due to the bioceramic sealer having certain expansion characteristics versus the shrinkage of the resin sealers [6]. So, in our opinion, the SC technique should only be used with bioceramic sealers by the expansion they exhibit. In addition, we thought by increasing the volume during the setting, it would be one of the factors that influenced the dentinal tubule penetration. In the study by Eymirli et al. [38] they studied one group with only sealer and they observed that the sealer had penetrated in the dentinal tubules by itself. For this reason, this one was only of several factors influencing sealer penetration.

Eid et al. [8] compared two different techniques CW and SC with two bioceramic sealers (HiFlow and Bio-C sealer®). They showed better diffusion levels for both sealers with CW than SC [8] in the middle compared to the apical third, as in our study. Yang et al. [47] evaluated two techniques, SC and CW with two bioceramic sealers (HiFlow, iRoot SP®) and one resin sealer (AH Plus®). In the dentinal tubule penetration area HiFlow/CW was significantly higher than in the iRoot SP/SC at apical level. They also exhibited the HiFlow/CW may have better sealing ability than the iRoot SP/SC technique in the apical third [47]. HiFlow with the CW had superior sealer penetration in the different thirds of the root canal than iRoot SP® with the SC. This increase of penetration may result in better apical sealing and improve the root canal filling [47]. This study offered results in line with our own with regard to major tubule penetration sealer with warm obturation techniques in all thirds. In our opinion a difference between the CW, VC and SC that could affect dentin tubules penetration was compaction with heat, the apical pressure and the flow of the sealer. According to one earlier study [20], HiFlow had higher flow than iRoot SP®.

The clinical relevance of our results may serve as a reference because the high ability of dentinal tubule penetration of the sealer with warm obturation techniques may improve the sealing action in the tubules, and therefore the antibacterial effect.

The blended use of HiFlow with the CW technique exhibited a good dentinal tubule penetration, so it is possible to achieve better apical sealing [47]. In addition, HiFlow was shown to have favourable biological properties and promoted expressions of osteo/cementogenic genes in human periodontal ligament stem cells [56]. These properties could make the blended use of the new bioceramic sealers with warm obturation techniques adequate for root canals with periapical periodontitis [47]. However, the best obturation technique for this material is still a matter under debate [8]. Features such as long-term clinical considerations, cellular responses, physicochemical properties, the use of warm obturation techniques with the new bioceramic sealers and antibacterial ability should be the subject of further research.

## 5 Conclusion

In conclusion, within the limitations of this study, the warm obturation techniques (CW and VC) showed more intratubular penetration of the calcium silicates-based sealer than the SC. The different gutta-percha tested in warm obturation techniques (NG and BG) were not influenced in the penetration area. For each type of gutta-percha and technique, dentinal tubule penetration was higher in the coronal section than in the apical section.

## Declarations

### Author contribution statement

Alberto Casino Alegre: Conceived and designed the experiments; Performed the experiments; Analyzed and interpreted the data; Contributed reagents, materials, analysis tools or data; Wrote the paper.

Susana Aranda Verdú: Conceived and designed the experiments; Analyzed and interpreted the data; Contributed reagents, materials, analysis tools or data; Wrote the paper.

Jose Ignacio Zarzosa López; Jorge Rubio Climent, Dr; Antonio Pallarés Sabater, Dr; Eliseo Plasencia Alcina, Dr: Analyzed and interpreted the data; Contributed reagents, materials, analysis tools or data; Wrote the paper.

### Funding statement

This research did not receive any specific grant from funding agencies in the public, commercial, or not-for-profit sectors.

### Data availability statement

Data will be made available on request.

### Declaration of interest’s statement

The authors declare no conflict of interest.

### Additional information

No additional information is available for this paper.
